# Computer-Assisted 3D Kinematic Analysis of All Leg Joints in Walking Insects

**DOI:** 10.1371/journal.pone.0013617

**Published:** 2010-10-26

**Authors:** John A. Bender, Elaine M. Simpson, Roy E. Ritzmann

**Affiliations:** Department of Biology, Case Western Reserve University, Cleveland, Ohio, United States of America; Freie Universitaet Berlin, Germany

## Abstract

High-speed video can provide fine-scaled analysis of animal behavior. However, extracting behavioral data from video sequences is a time-consuming, tedious, subjective task. These issues are exacerbated where accurate behavioral descriptions require analysis of multiple points in three dimensions. We describe a new computer program written to assist a user in simultaneously extracting three-dimensional kinematics of multiple points on each of an insect's six legs. Digital video of a walking cockroach was collected in grayscale at 500 fps from two synchronized, calibrated cameras. We improved the legs' visibility by painting white dots on the joints, similar to techniques used for digitizing human motion. Compared to manual digitization of 26 points on the legs over a single, 8-second bout of walking (or 106,496 individual 3D points), our software achieved approximately 90% of the accuracy with 10% of the labor. Our experimental design reduced the complexity of the tracking problem by tethering the insect and allowing it to walk in place on a lightly oiled glass surface, but in principle, the algorithms implemented are extensible to free walking. Our software is free and open-source, written in the free language Python and including a graphical user interface for configuration and control. We encourage collaborative enhancements to make this tool both better and widely utilized.

## Introduction

Students of animal behavior have long recognized the importance of high-speed videography to understanding the mechanisms of locomotion. These investigations, tracing their roots to the famous horse photography of Eadweard Muybridge in the 1870s, have remained in the mind of ethologists to the present day. As high-speed cameras have improved and become more widely available, more and more insights have been achieved in animal behavior and neuroscience (e.g., [1], [2], [3], [4], [5]). However, at present, the bottleneck in video analysis is human time. The majority of analysis consists of an operator manually selecting points of interest in frame after frame of video, digitizing or extracting an animated sequence of motion. Not only is this tedious and time-consuming, it is also subjective. Moreover, the onerous nature of digitizing video effectively limits the application of this technique to relatively simple problems. Ideally, one would like to be able to analyze very complex behaviors, such as those in which multiple joints of multiple appendages play through a concert of kinematic patterns. It seems as if the ever-increasing speed of desktop computers should have something to offer.

Unfortunately, today's computer-vision algorithms are often very sensitive to image quality and initial conditions. The film and gaming industries make extensive use of the best motion-capture technology, but the procedures are still finicky, expensive, laborious, and worse, sometimes invasive. For animals smaller than a dog or cat, placing light-emitting or reflecting markers on the body can become restrictive to movement and behavior. For insects, a model system of choice for many studies in neuroethology, the problem is rendered still more difficult because their bodies are much smaller, giving the experimenter a choice between low spatial resolution or restricting the animal in space. Tracking the body position and orientation of moving insects is already feasible (for example, Fry et al. did this in real time during flight [6]), but more complex investigations of neuromuscular control involving appendages still require additional technological innovations. Many walking insects have long legs that may move only a small amount during each step, so tracking algorithms must work well in space poorly resolved by the cameras.

Early studies on walking insects restricted motion analysis to one dimension, delimiting strides by the anterior/posterior position of the foot [7], [8], [9]. Even this measurement is complicated by the fact that insects have six legs moving together in a small volume of space, so from a ventral view, the feet often occlude each other when one is touching the ground (in the stance phase of stepping) while another foot is in the air (in the stride phase). The insufficiency of measuring only the foot's anterior/posterior motion is most obvious when the animal turns, as the legs then no longer move parallel to the body axis. An additional stumbling block arose when investigators realized that the anterior and posterior extreme positions of the feet (AEP and PEP, respectively) are not synonymous with the beginning and end of the stance phase. In fact, the foot starts to move backward before it touches the ground at the beginning of stance and also lifts off the ground before starting to move forward at the beginning of swing [2]. Presumably, both the forward/backward movement and the load-bearing stance period have implications for the animal's neurons and muscles, so at least two dimensions of motion must be analyzed.

The problem is more difficult when one considers the movements of joints that make up an appendage, in order to relate movement to motor neuron activity. The joints on the hind legs of a walking cat, a jumping locust, or even a sprinting cockroach may be adequately described in 2D. However, in some overactuated systems, multiple configurations of the joints could lead to identical positions of the endpoints. For example, the human arm can reach out to grasp an object at the same position in space using many different combinations of shoulder, elbow, and wrist angles. To understand how those joints are controlled during the reaching movement, each joint angle must be measured at all times. This cannot be accomplished in 2D; a 3D analysis of motion is required. Likewise, although the middle and hind legs of a cockroach may be effectively planar during forward walking, the front legs are much more complex. Initial 3D motion studies of the cockroach explicitly avoided the front leg joints as too difficult to analyze, though one study did describe a single stride. However, the front legs are critical in steering and adjustments to obstacles in most, if not all, insects [10], [11], [12], [13]. Even a detailed, 3D analysis of the front legs alone is less than ideal, since at least the middle legs of many insects can perform multiplanar movements during turning. Detailed patterns of joint coordination in an insect can only be revealed by looking at all six legs simultaneously, but simultaneous 3D analysis of all the joints on all six legs using high-speed video is a daunting task.

We describe here a new method to extract the 3D motion of each joint on all six of an insect's legs by tethering an animal and inducing it to walk in place on a sheet of oiled glass (after [14], [15]). Placing small dots of reflective paint near the leg joints, we were able to capture the 2D motion of 26 unique points at high spatiotemporal resolution using two calibrated cameras. We describe an algorithm that assists an operator in extracting the 3D positions of each of these points, finally allowing for a detailed, neurally relevant description of many walking steps.

## Materials and Methods

### Animals

**Figure 1:**
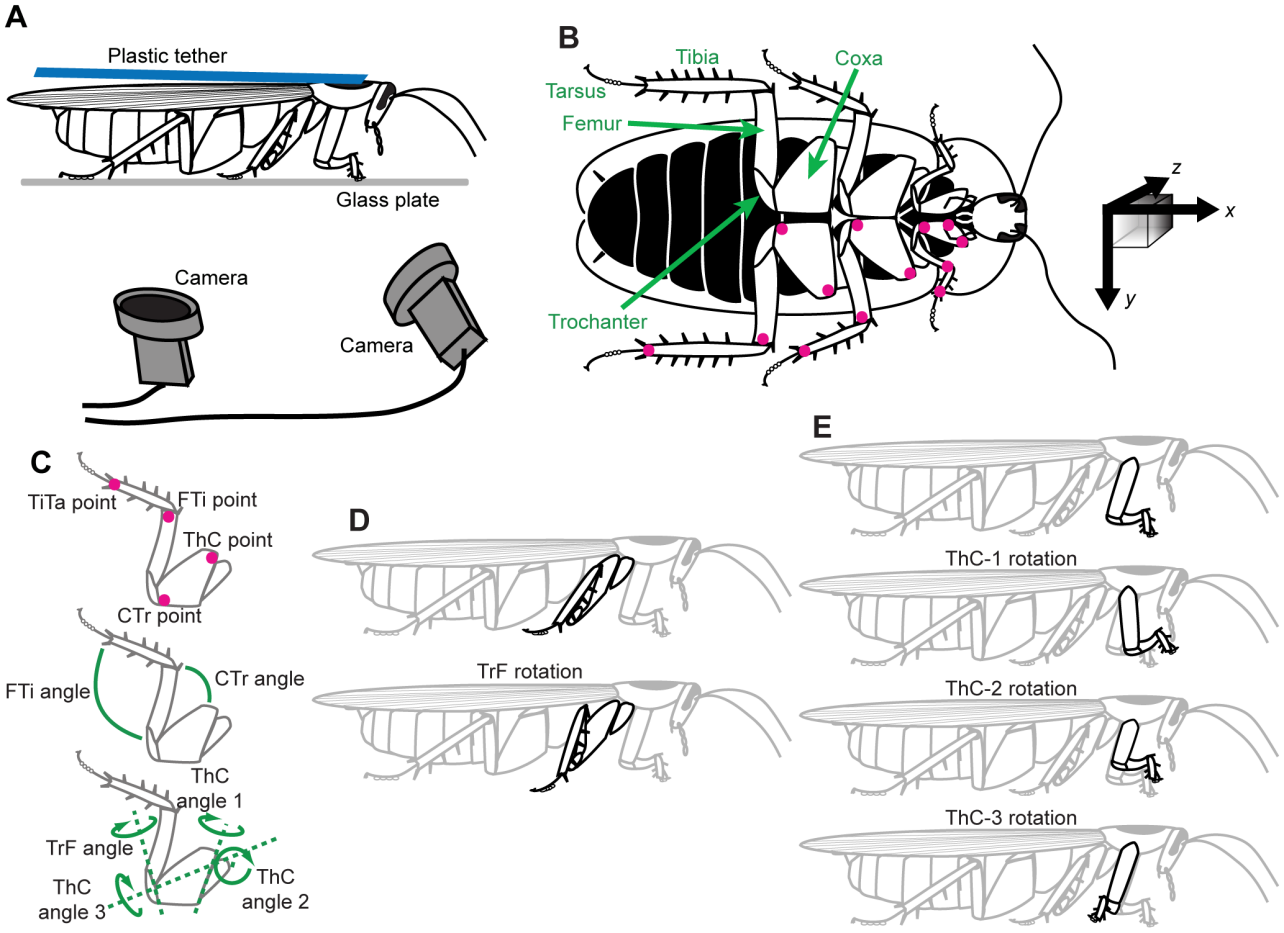
Experimental setup. (A) The recording configuration. A cockroach was glued to a flexible tether and walked in place on a plate of oiled glass. One camera was slightly to the front of the animal and the other viewed its ventral surface through a mirror. (B) Ventral view of a cockroach, *Blaberus discoidalis*, with the body colored black for contrast. The colored dots on the legs indicate the points which were marked and then tracked by our software. The black arrows at right indicate the coordinate system used in this analysis, with the *z*-axis extending into the page (shaded cube added to indicate depth). The *x* and *y* vectors shown would be approximately 1 cm in length. (C) Details of digitized points and definitions of joint angles. (D) Reduction of the trochanter-femur (TrF) joint results in lowering the foot toward the substrate. (E) The thorax-coxa (ThC) joint has three rotational degrees of freedom in the front leg; in the middle and hind legs, only the first two degrees of freedom are actuated.

We used adult female cockroaches, *Blaberus discoidalis*, from a laboratory colony. Animals were removed from the colony and anesthetized using ice. We tethered each cockroach by gluing a small (1×7 cm) piece of plastic to the dorsal surface of the pronotum (the cuticular shield above the prothorax). This tether allowed a small amount of dorsal-ventral flexion, which is important in eliciting walking behavior in this species. We applied a small amount of white paint to the leg joints (Fig. 1A) to aid in locating these points in the video images. During experiments, the tether was positioned such that the dorsal surface of the pronotum was in the range of 1.2–1.8 cm above the surface of a glass plate, corresponding to a normal walking pose for animals of different sizes. The glass plate was prepared with several drops of transparent microtome oil (Lipshaw, Detroit, MI, USA), spread evenly over the working area.

### Video capture and calibration

We positioned two synchronized, high-speed, digital video cameras (MotionScope, Redlake Imaging, Morgan Hill, CA, USA) beneath the glass plate to obtain different ventral views of a walking cockroach (Fig. 1B) illuminated with infrared light. Grayscale images were captured with an image resolution of 320×280 pixels, corresponding to an approximate real-world resolution of 30 pixels cm^−1^. Video frames were acquired at 500 fps in sequences of 8 s at a time (4096 frames). These pairs of movies were saved to disk, and only trials or portions of trials (minimum 5 s) during which the animal appeared to perform limb motions corresponding to forward, straight walking at a constant step rate were used in subsequent analysis.

Because they are two-dimensional, points in a camera image are ambiguous with respect to depth. If the position of the camera is known, however, 2D image points become 3D rays extending from the camera into space. If multiple viewpoints are available, the 3D position of an object can be calculated by triangulation, because if the 2D image points represent the same 3D object, the camera rays should intersect. The distance between the rays at the point where they come nearest to intersecting (the triangulation error) can be a useful measure of confidence in the correspondence of those two image points. We calculated the positions of our cameras with an iterative camera calibration algorithm [16] implemented in the open-source computer vision software library OpenCV (http://sourceforge.net/projects/opencvlibrary/, version 1.1), applied to a calibration jig of known geometry. One way to quantify the accuracy of a camera calibration is by retrospectively comparing the pixel locations of the points on the jig with their expected positions calculated from the camera calibration. The difference between the actual image positions and the estimates is called the reprojection error, and ranged from 0.3 to 0.9 pixels in our system. Another error metric is the retriangulation error, which uses pairs of 2D pixel locations from the two cameras to estimate the 3D positions of those points. The average difference between those triangulated positions and the known, 3D positions of the calibration points was around 0.3 mm. Calibration images were acquired after the glass plate was oiled. 3D triangulation is optimal when the cameras are orthogonal, but since a cockroach's legs are almost entirely underneath its body, we were forced to position both cameras below the animal. This led to relatively larger stereo reconstruction errors in the *z*-dimension (vertical with respect to the animal) than in the *x*- or *y*-dimensions. For any given 3D point within our tracking volume, translating its position into camera coordinates (pixels), rounding, and triangulating the corresponding rays gave reprojected *x*- and *y*-coordinate errors no greater than 0.15 mm and *z*-coordinate errors less than 0.3 mm.

### Image processing

**Figure 2:**
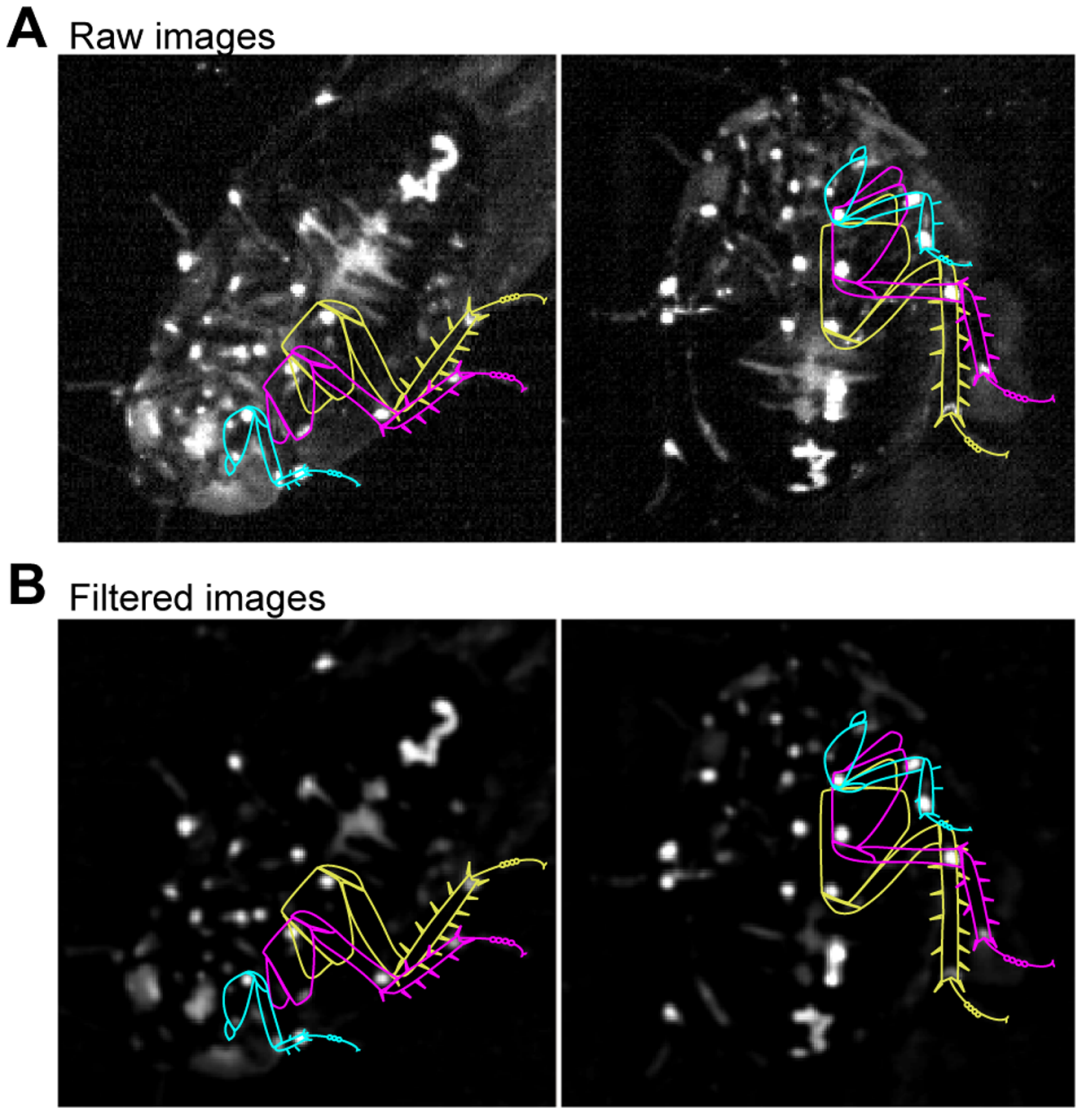
Image preprocessing. Leg outlines for the left leg are drawn as an aid to the reader.

Before tracking, our software processed each video image to increase its signal-to-noise ratio (Fig. 2). First, an average background image was calculated for the entire movie. 100 frames, spaced evenly throughout the movie, were filtered using a Gaussian filter with a standard deviation of about 5 pixels, and the mean of these filtered frames served as the background image. Each video frame was modified by subtracting this background image and then applying a median filter with a width around 5 pixels. This processing sequence subjectively seemed to produce the cleanest results in our recording setup, in the sense that the white, painted points were highly salient and most other image features were darkened. The exact widths of the filters were varied slightly from day to day as the lighting was altered. As can be seen in Figure 2, filtering somewhat reduces the precision with which the point positions can be estimated, but also enhanced accuracy in this case, resulting in fewer required manual corrections (see below). The software allows the user to easily choose the desired level of compromise between precision and accuracy by adjusting the filter settings. The precision lost due to the settings given here is reflected in our error estimates (see below).

### Tracking

In order to accurately extract the 3D positions of the animal's leg joints throughout the sequence of pre-processed video images, our software used several complementary strategies. First, an initial position for each of the joints in one frame was defined by the user. Automatic tracking (see below) proceeded both forward and backward through the movie from that user-selected point in time. When the program had estimated positions for each joint at each point in time, the user was allowed to scroll through the movie and view the results. If a frame contained an obvious mismatch between the position of a leg point and the tracked estimate of that point, the user had the option to update the tracked position. Once the leg positions were redefined by the user in a frame, that frame was used as another initialization point, and automatic tracking again worked forward and backward in time from there. This tracking update never extended more than halfway to a previously user-defined frame, to avoid overwriting positions which were already verified. For the data presented here, the user typically updated one or more points on <3–5% of the frames in each movie, but could easily annotate more frames if more accuracy was necessary.

The automatic tracking algorithm used the known (or previously calculated) positions of the leg joints in one frame to locate the corresponding positions in the next frame. This process took advantage of the oiled-plate tether and the known 3D geometry of the legs – specifically, that the body (and therefore the thorax-coxa, or ThC, joint) does not change position during the experiment, that lengths of the leg segments should be fairly constant in time, and that more distal points can move more quickly than more proximal points. Given this knowledge, we tracked each leg separately, beginning from the ThC point and working distally.

**Figure 3:**
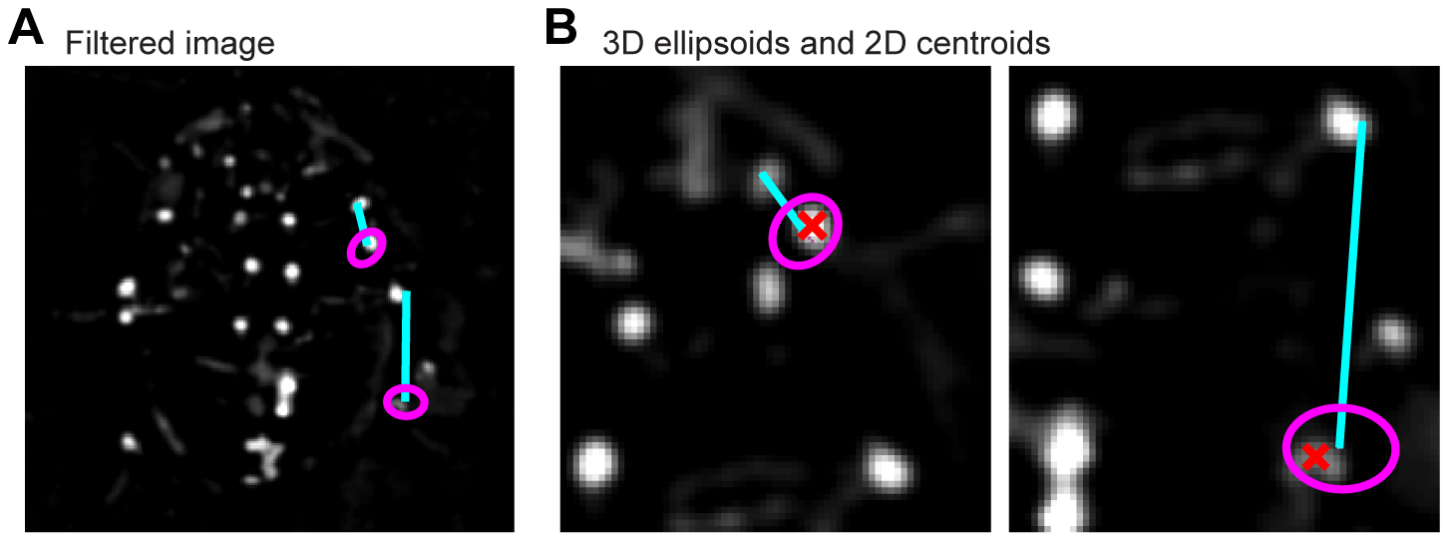
Point-extraction algorithm. (A) Video images have the average background subtracted and are then filtered to amplify and localize the white dots painted on the cockroach's legs. The colored lines approximate the areas shown in greater detail in panel B, though from different images. (B) Proceeding from proximal to distal points on each leg, a 3D ellipsoid is defined based on the last known position of each point, with its short axis along the line between the point and the next-most-proximal point on the same leg. This ellipsoid is projected onto the image from the camera, and the intensity-weighted centroid of the pixels within the resulting ellipse defines the new estimate of that point's 2D position. The blue lines are the ellipsoid axes. The pink ellipses are the 2D projections of the corresponding ellipsoids constraining the search area, and the red Xs show the centroids of the ellipses.

We utilized the very high frame rate to make the best *a priori* estimate of a point's position at time *t* the same as its known position at time *t*−1 (or *t*+1). Therefore, the software searched for each point within a three-dimensional ellipsoid centered at the last known position of that point (Fig. 3). This ellipsoid was actually a flattened sphere, with a circular cross-section along one axis. The ellipsoid was rotated such that its short axis was the same as the axis of the leg segment along which the current search was conducted. This segment extended from the already-estimated position of the next most proximal point to the last known position of the current point. The shortened axis of the ellipsoid represented the fact that the length of the leg segment was unlikely to grow or shrink, while the circular off-axis search area suggested a complete lack of knowledge about the possible rotation of the leg joint proximal to the current point. A more ideal search volume would be something like a piece cut from a hollow sphere, but the ellipsoid simplification is more geometrically tractable. The lengths of the ellipsoid's axes were a user-defined variable and scaled exponentially with increasing distance along the leg. This scaling factor was also user-defined, but the short axis of the ellipsoid always had half the length of the long axes. For the ThC joint, the ellipsoid was simply a small sphere, since no movement was expected.

The 3D ellipsoid was then projected onto the image plane of each camera, serving as a 2D elliptical search area within the corresponding movie frames. Since the points of interest are white against a dark background, the new 2D position was easily estimated as the brightness-weighted centroid of the pixels inside the ellipse, calculated from the first central moments of the image. The two 2D positions were triangulated to compute a new 3D position for the leg point. If the brightness of the centroid pixel in one image was less than half the median brightness of the image, a new 3D ellipsoid was generated with a larger radius, for that camera view only. If the new centroid was still too dim, or the triangulation error was above a user-defined threshold, or the new 3D position caused the leg segment to change length by more than another threshold factor, then the point was assumed to be lost (possibly occluded). In this case, the leg point was reverted to its last known 3D location.

**Figure 4:**
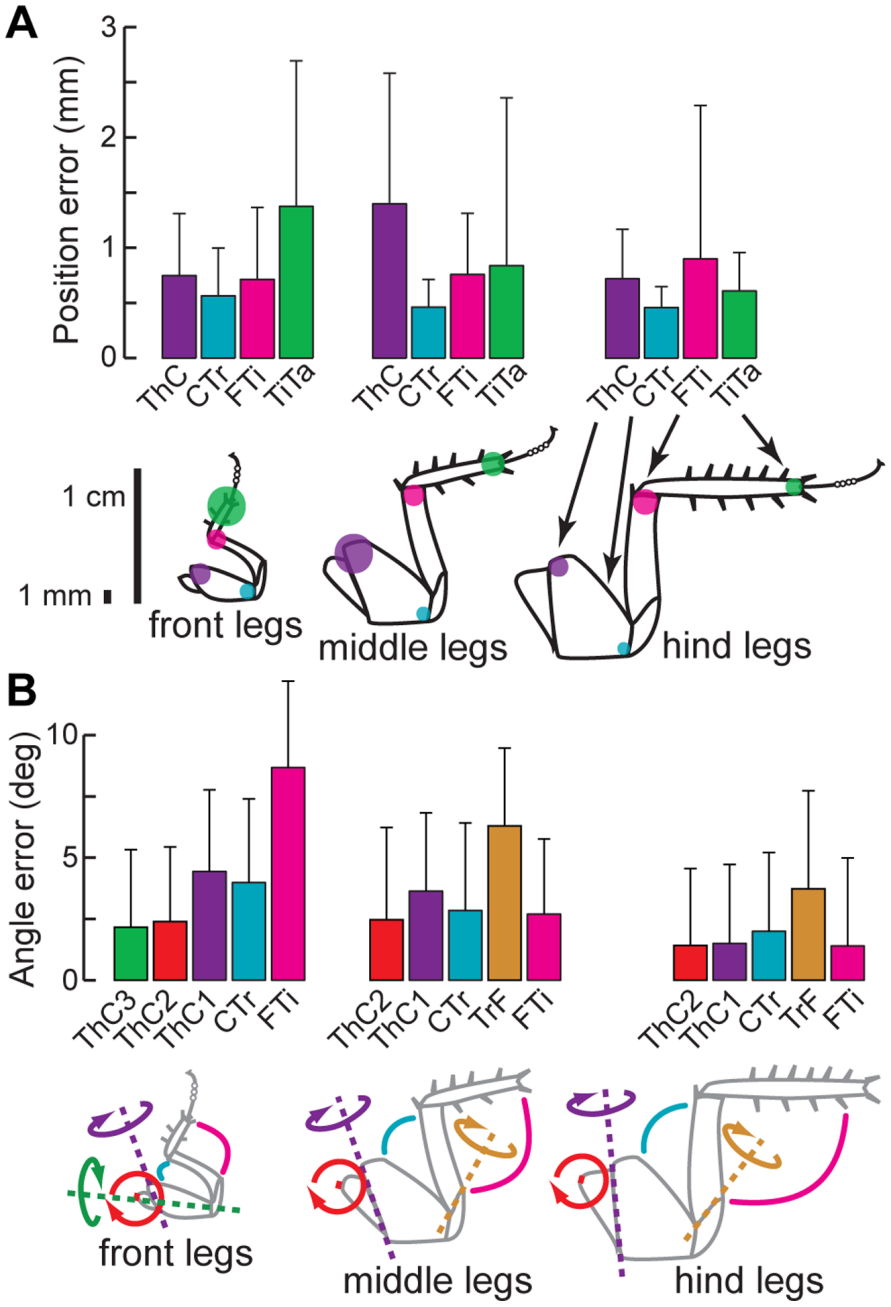
Our user-assisted digitization process yields results with accuracy comparable to manual digitization. (A) Each colored bar indicates the mean and standard deviation of the 3D Euclidean distance from the manually digitized point to the same point extracted by our software, for a single walking bout (4096 frames). The colored dots on the scale drawings of the legs have a radius corresponding to approximately the average positional error. (B) Each bar shows the mean and standard deviation of the absolute joint angle error. Joint angle errors were distributed log-normally.

No additional assumptions were made about the behavioral patterns of leg movement. Some such constraints could be potentially valuable as tools for more effective tracking (e.g., the relative positions of the joints during stride versus during stance, the joints' rotation axes, the general forward-back temporal pattern of motion, or the correlations between legs). However, we chose not to make use of these sources of information to avoid fitting our data to our assumptions, some of which remain to be tested explicitly. We quantified the reliability of our tracking method by manually digitizing one 8 s trial, or 106,496 three-dimensional positions. Our software's averaging tracking error relative to the human “gold standard” was generally less than about 1 mm, except for the TiTa points of the front legs, which were slightly worse (Fig. 4). These translated into joint angle errors of 2–4°, except for somewhat larger errors for the FTi joints of the front legs, caused by the TiTa tracking errors. Overall, this seems to be a reasonable compromise for digitizing each movie in hours instead of days, and is of the same order as previous approaches [17].

### 3D data processing

**Figure 5:**
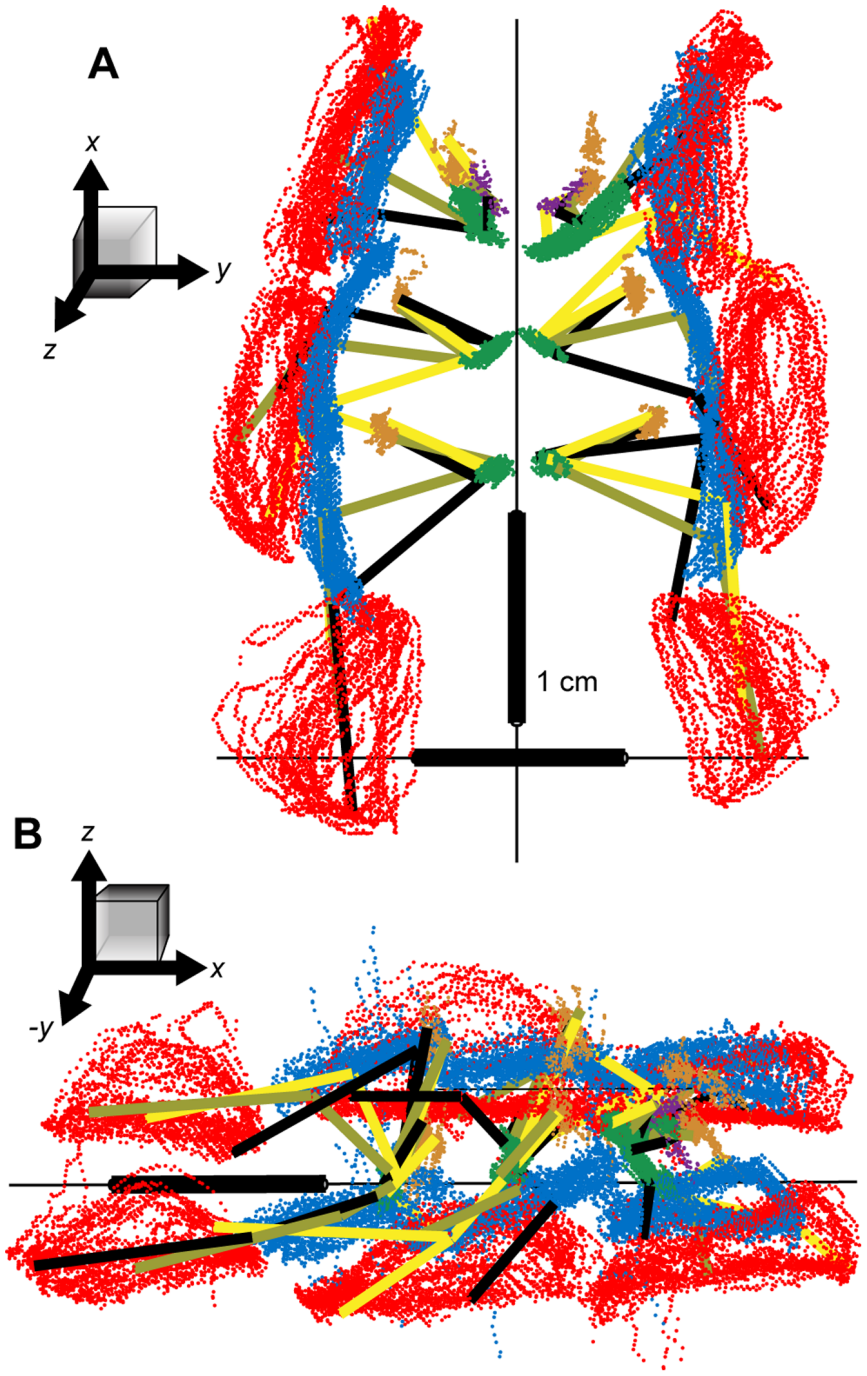
Raw 3D positions of the tracked points through a single, 8-second bout of walking. (A) A ventral view. (B) View from the animal's right and slightly above the substrate. In both panels, the red points indicate the positions of the tibia-tarsus (TiTa) joint; the blue points: the femur-tibia (FTi) joint; green: the coxa-trochanter (CTr) joint; orange: ThC joint; and the purple points are the extra dots placed on the coxae of the front legs to aid in determining their rotation. The black, olive, and yellow line segments connect the points of each leg as they appeared in selected, synchronous video frames. The cockroach did not appear to be walking precisely straight forward during this trial, as most obviously indicated by the left-right asymmetry in the front leg TiTa positions.

**Figure 6:**
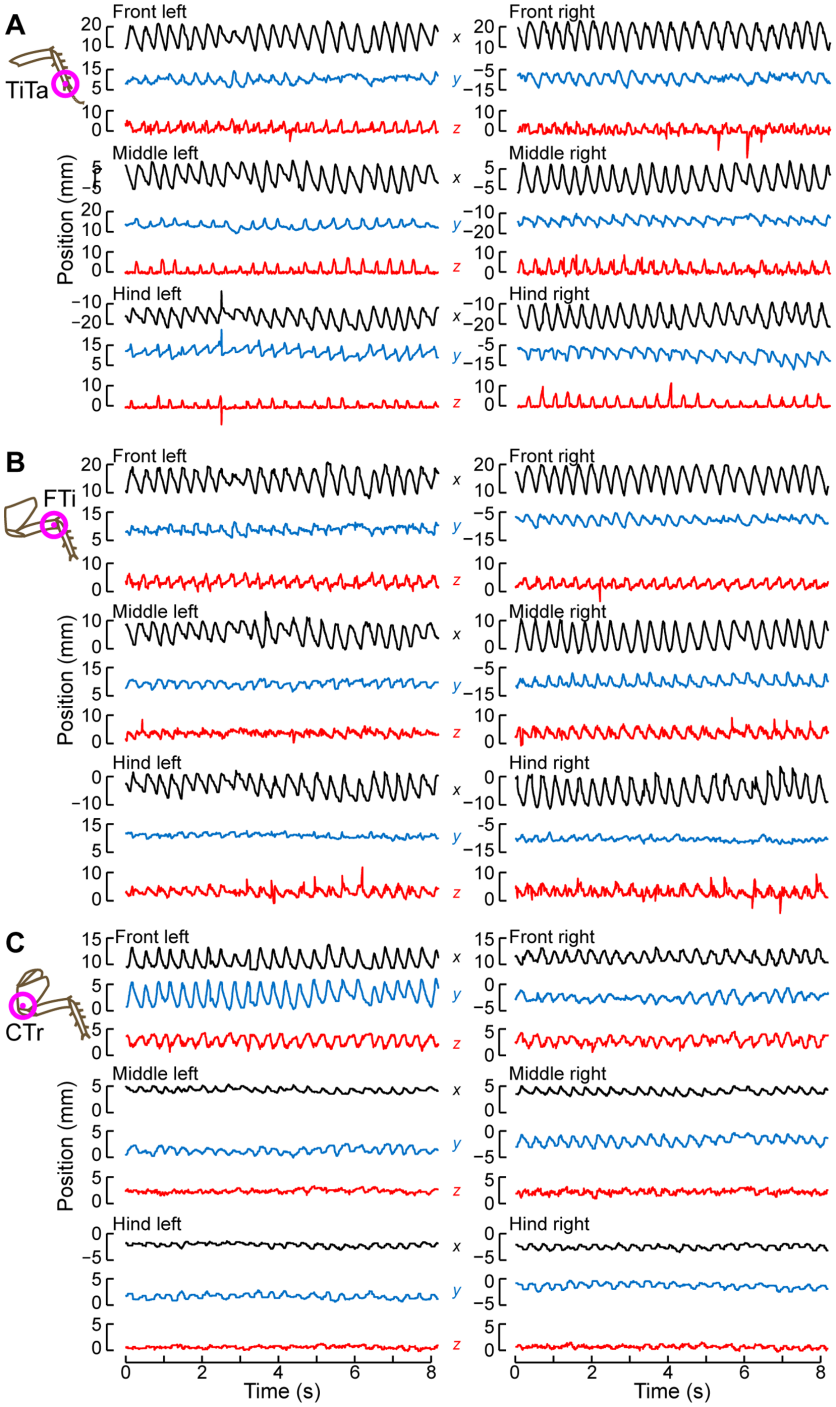
Time-series of leg positions during the same bout shown in **Fig. 5**. The black traces show the *x* (forward-back) positions of the points; red: *y* (right-left) positions; blue: *z* (up-down) positions for (A) the TiTa point, (B) the FTi point, and (C) the CTr point.

After all 3D points were extracted and confirmed by a user, the points required rotation from a variable coordinate system defined by the calibration jig into a common frame of reference. We defined our coordinate system such that the *x*-axis pointed toward the animal's head, the *y*-axis was to the left, and the *z*-axis extended up from the substrate, with the origin near the geometric center of all the data points (Fig. 5). For each movie, first, the orientation of the *z*-axis was determined under the assumption that the median value of all the tibia-tarsus (TiTa) points was on the ground (i.e., at *z* = 0). We therefore calculated a normal vector to the *xy*-plane as the average of the cross products of several leg-to-leg direction vectors, and defined this vector as the *z*-axis. Specifically, if *l*_1_..*l*_6_ are the median values of the raw TiTa points for legs 1 through 6 (where 1 is the right front and 2 is left front), then the *z*-axis was defined as
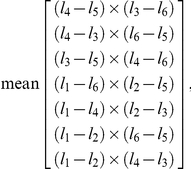
normalized so that the *z*-value of each cross product had the same sign. Second, the long body axis was estimated as the best-fit line through three points: the mean of all the coxa-trochanter (CTr) point positions in both middle legs, the mean of the CTr points in both rear legs, and the mean of the ThC points in both front legs. We rotated the new coordinate system around the *z*-axis until the *x*-axis was parallel to this body axis, with the ThC joints of the front legs having a larger *x*-value than the rear legs. Finally, the coordinate system was shifted along the *y*-axis by the mean of the *y*-values of the median CTr point for each leg. These transformations left the 3D data for each movie in a comparable coordinate system for further analysis. Figures 5–6 show the 3D positions extracted from one movie, rotated into this reference frame and plotted in either space or time. The apparent outliers in these plots represent tracking errors not corrected by the user at this level of detail. More time spent by a user would certainly result in a reduction of the error rate and magnitude, but with diminishing returns on the time invested. The data shown here correspond to a compromise we deemed acceptable for our analyses, and resulted in the error rates shown in Figure 4.

**Figure 7:**
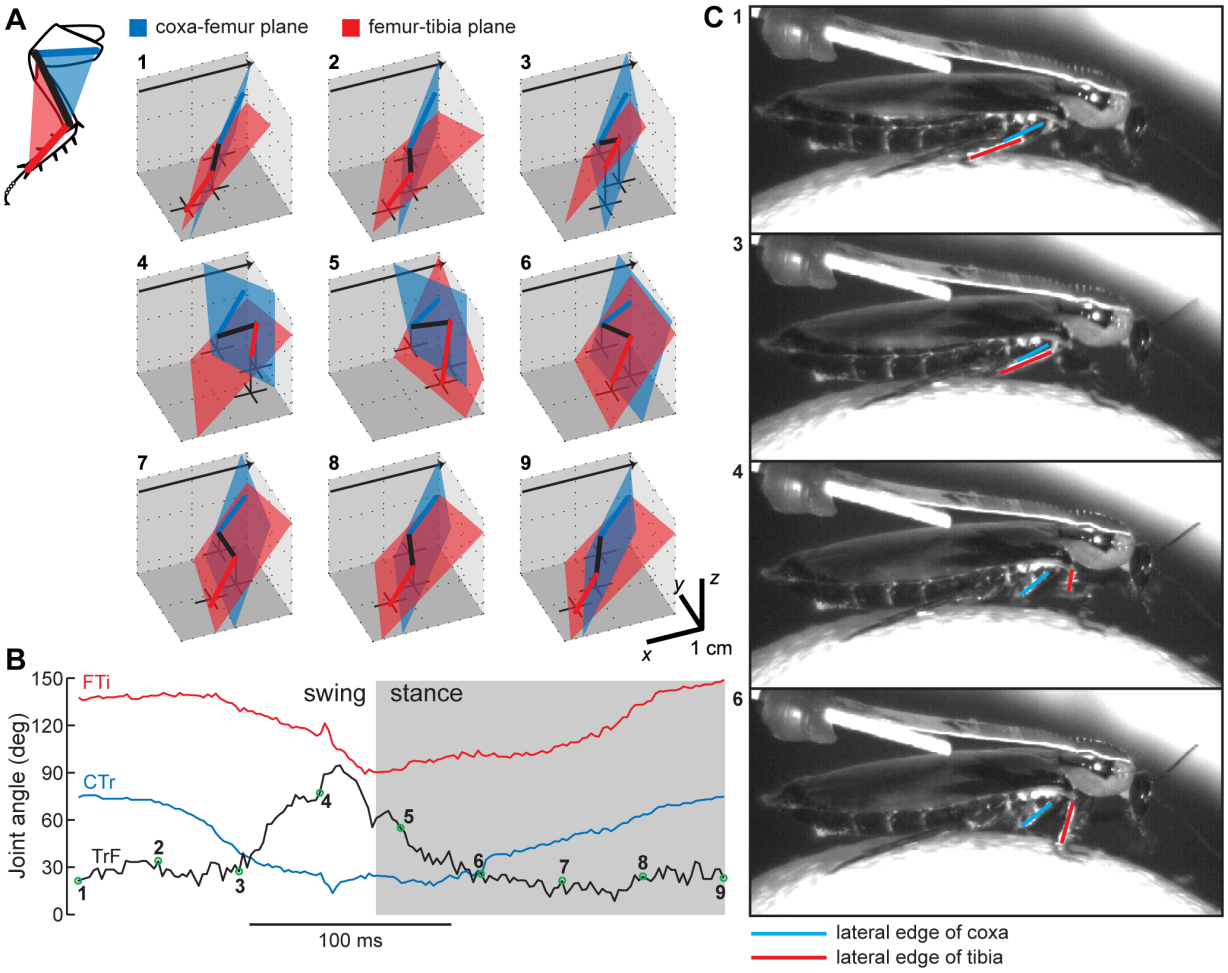
The measurement and action of the TrF joint. (A) For one stride by the right middle leg, the motion of the leg is depicted in 3D as a stick figure. Leg segments and planes are colored as in the inset (top left). The plane formed by the ThC-CTr-FTi points is blue, and the plane formed by the CTr-FTi-TiTa points is red. The TrF angle is defined as the angle between these two planes. The black crosses show the projection of the CTr, FTi, and TiTa points onto the ground plane, with a vertical black line connecting each cross to its corresponding joint. The black arrows point toward the animal's head. (B) The FTi, TrF, and CTr joint angles over the same stride plotted in panel A. The 9 colored points correspond to the 9 stick figures in A. (C) High-speed video of one stride by a cockroach walking on an air-floated Styrofoam ball. The lateral edges of the tibia and the coxa were painted with white paint to increase contrast, and are overlaid with red and blue lines here, respectively. The 4 video frames were chosen to approximate the step phases shown by the appropriately numbered points in panels A and B. The red and blue lines are parallel in the top two frames, and rotated in the bottom two frames. This rotation corresponds to reduction at the TrF joint.

The joint angles for the femur-tibia (FTi) and CTr joints were calculated using triplets of neighboring points, specifically the TiTa-FTi-CTr triplet and FTi-CTr-ThC triplet, respectively. Measuring the angles of the trochanter-femur (TrF) and ThC joints was not as straightforward. The TrF joint appears to be fixed on the front legs of *B. discoidalis*, but for the middle and hind legs, we estimated the TrF angle as the angle between the planes defined by the TiTa-FTi-CTr points and the FTi-CTr-ThC points (Fig. 7). The middle and hind legs are normally two-dimensional except when the TrF joint actively rotates (reduces) the femur relative to the coxa and trochanter.

The ThC joints of the hind and middle legs have two degrees of freedom, anatomically termed adduction/abduction and promotion/remotion, though because of the limbs' posture, the promotion/remotion movement could be considered depression/levation. The promotion/remotion angle, herein called ThC_1_, was calculated as the angle within the *xz*-plane of the CTr-ThC segment relative to the vertical (*z*) line through the ThC point. The adduction/abduction angle (ThC_2_) was the angle of the CTr-ThC segment, rotated by ThC_1_ and projected into the *yz*-plane, relative to the vertical line through the ThC point. The ThC joint of the front legs also contains a third degree of freedom (ThC_3_): rotation about its long axis. An extra point was digitized midway along the coxa to define a coxal plane. The magnitude of the ThC_3_ rotation was approximated by taking a plane, parallel to the *yz*-plane, which would define ThC_3_ = 0 if ThC_2_ = 0 and ThC_1_ = 0, and rotating that plane first by ThC_1_ and then by ThC_2_, in keeping with the kinematic order of these joints. The intersection of this rotated plane with the coxal plane was the long axis of the coxa. The orientation of of the coxal plane relative to the rotated plane determined ThC_3_. Mathematically, if 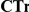 is defined as the 3D coordinate vector of the CTr point (etc.), this kinematic chain is formalized as
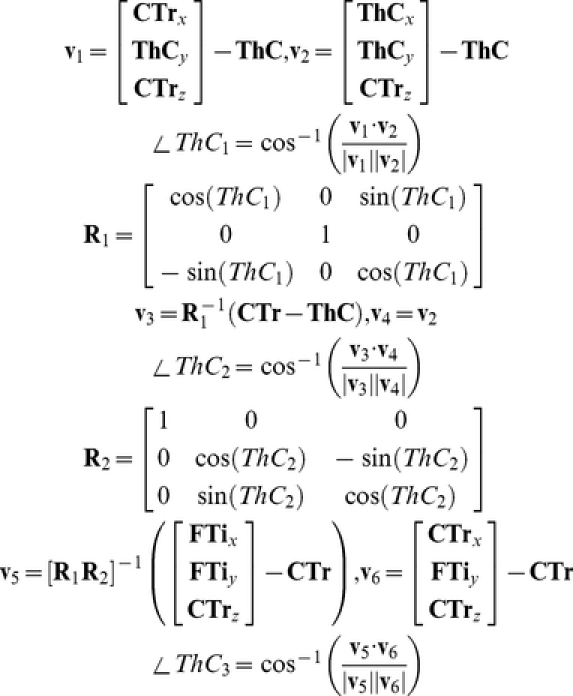
where **v_1_**..**v_6_** are variables defined for convenience.

### Stride timing

**Figure 8:**
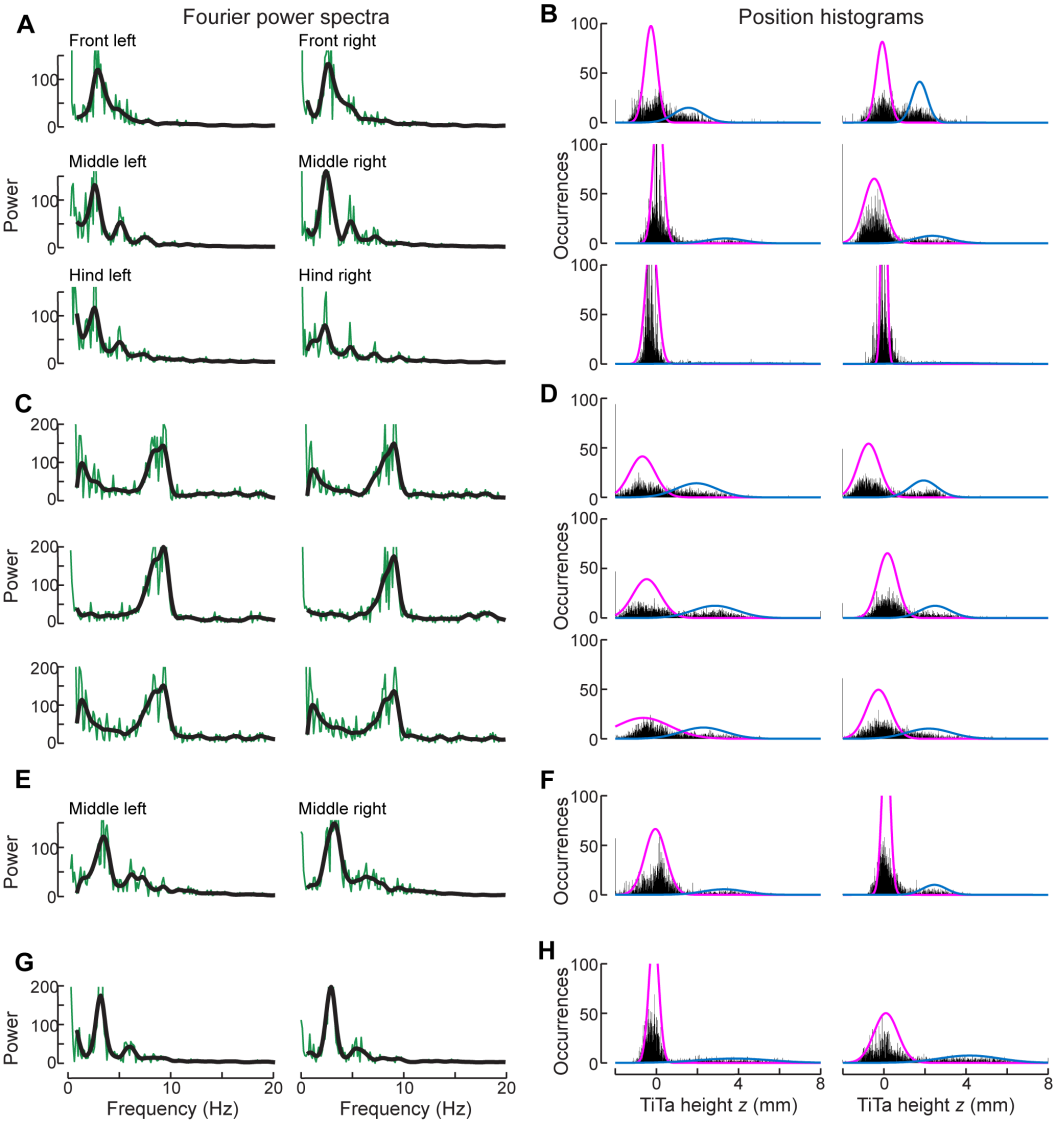
Parameters used to calculate stride timing. (A) Fourier power spectra of the legs' *x*-positions, for the same walking bout shown in Fig. 5. Green: raw amplitude; black: filtered amplitude. The peak frequency in the black curves is the initial estimate of the stepping rate by each leg. (B) Histograms of the *z*-positions of the legs' tibia-tarsus (TiTa) joints during the same walking bout. Each histogram is fit with a 2-component Gaussian mixture model (colored traces), with the mean and variance of the lower-valued cluster (pink) used to help determine whether or not the foot was touching the substrate. (C,E,G) Power spectra for other trials, each with a different average step rate (note the different frequencies of the peak power). Panels E and G show the middle legs only. (D,F,H) *Z*-histograms for the same bouts.

We first estimated the step frequency for each leg as the peak in a discrete Fourier transformation of the time-series data for the TiTa point's *x*-coordinate (*i.e.*, the frequency at which the foot moved forward and backward). The mean of the peak frequencies for each leg was chosen as the initial estimated frequency *f* for the whole trial (Fig. 8A). We then applied to each leg's *x*-coordinate data a zero-delay, fourth-order, Butterworth filter with a lowpass cutoff at 2*f*, using this filtered data to estimate the derivative by the central difference method. The times at which the derivative changed sign provided initial estimates for when the leg switched from moving forward to moving backward or vice versa. We then used one of two algorithms to find the exact times of stride transitions based on these estimates.

**Figure 9:**
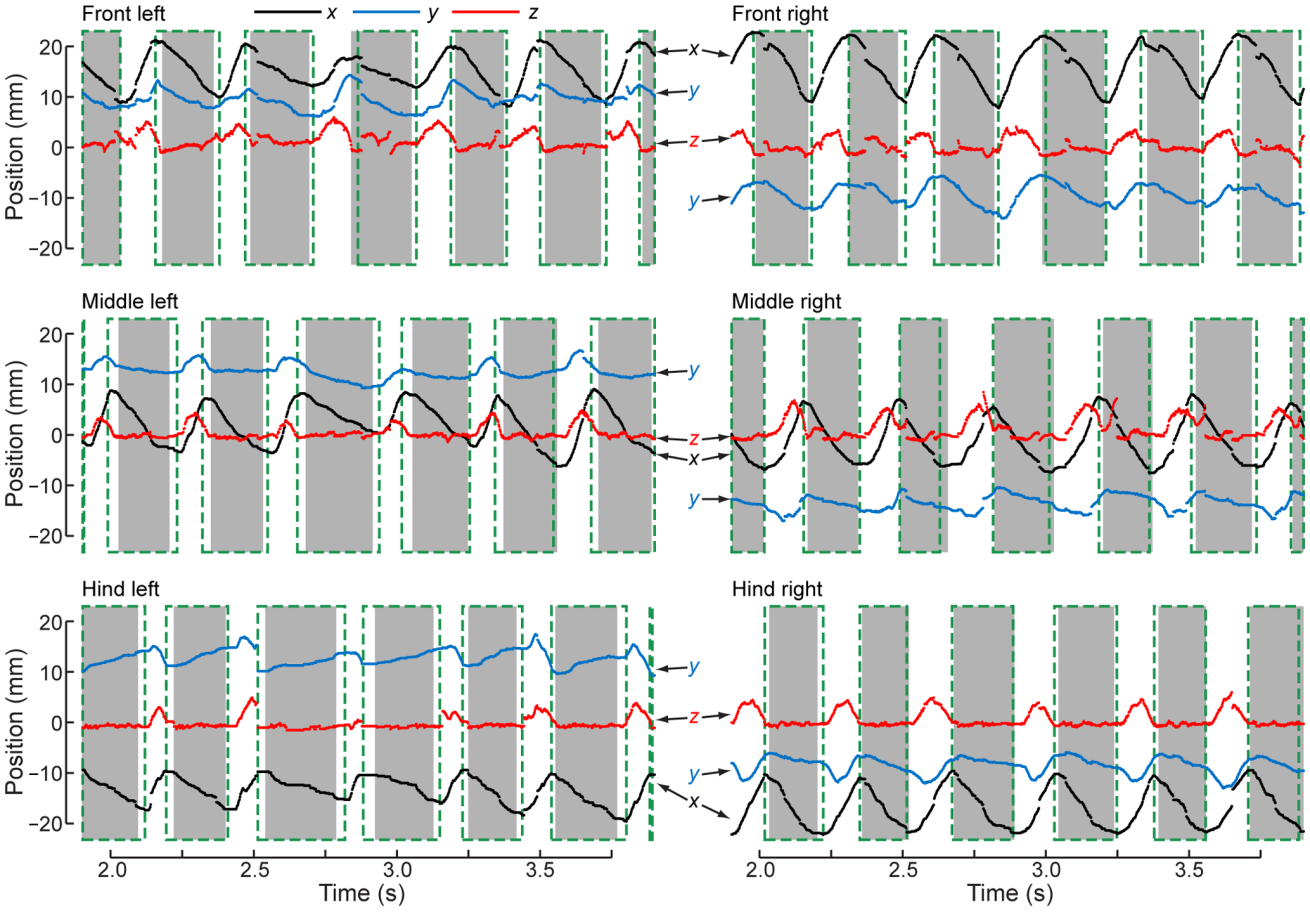
Calculation of stride timing. (A) Enlarged view of a portion of the walking bout shown in Fig. 5. Although it is not obvious, the *x*-, *y*-, and *z*-positions of the TiTa points (shown in black, red, and blue, respectively) are displayed here as points rather than lines to demonstrate the discretization due to the digital video capture. Discontinuities typically occurred when the forward and backward tracking intersected (see Methods). The gray boxes denote the stance phases of each leg, as defined by the leg's *z*-coordinate. The dashed, green boxes show how the stance phase calculation changes when the foot's anterior and posterior extreme positions (AEP and PEP, respectively) are used to determine stride timing.

We first calculated stride transitions using the one-dimensional AEP and PEP within a short time window (width 1/4*f* s, generally 25–125 ms) around the estimates computed from the *x*-coordinate's derivative. We then made a second, complementary measurement of stride transitions including the *z*-coordinates of the TiTa points (i.e., their height above the substrate). For this, we roughly clustered the *z*-coordinate values into “swing” and “stance” bins by fitting a Gaussian mixture model to the distribution of *z*-values using the *k*-means algorithm (Fig. 8B). The mean (μ) and standard deviation (σ) of the cluster with the smaller *z*-value were used to represent values which occurred primarily during stance, when the foot was touching the glass plate. These values are not the same for each leg because of inconsistencies in the placement of the painted dots and small uncertainties in the coordinate transformation. Additionally, the histograms do not appear to be fully described by two Gaussians, but these fits were typically sufficient to distinguish ground contact. In the same time window (1/4*f* s) around the *x*-derivative estimates, we redefined the beginning of swing as the earliest time at which the *z*-value went above μ+σ and the beginning of stance as the latest time when the z-value went below μ+2σ. The AEP/PEP and *z*-value metrics yield similar but slightly different times for each stride (Fig. 9), which is not due to imprecision but because the legs do, in fact, begin moving backward before they touch the ground at the beginning of stance and continue moving backward even after they leave the ground at the beginning of swing [2].

### User interface

Because the largest contribution of this method is the software inteface for tracking, we made every effort to make the graphical user interface as user-friendly as possible. It is entirely open-source, in contrast with MATLAB software tools, and platform-independent, built in Python using the WxWidgets library. The main window is very small, consisting only of a “Play” button with speed adjusters and a series of menus. Once a movie is loaded, an additional large window opens for each camera view. The user selects “Initialize tracking” from the “Track” menu, and is then led through the process of camera calibration and selecting initial points in the video by clicking on them. Tracking feedback is always provided by changing the size of the displayed points. Large points indicate large errors in tracking, usually as a result of the tracked point wandering separately in the two camera views. Points are selected by clicking them and deleted by either double-clicking or using a hot key. Another mouse click replaces the point, and a context (right-click) menu allows other actions including “Undo”. After a point has been moved, if the user tries to change frames to a different time in the movie, he/she is first forced to “Update tracking”, which proceeds as described above. Every tracking update automatically saves the annotation data. The user also has the option to view whether the current frame was previously user-modified, and how far away in each time direction is the nearest user-defined frame. Calibration, display, and tracking parameters are set using a pop-up “Settings” window that is organized using tabs and contains all of the numerous user-modifiable variables. The user can choose to view the raw, filtered, or annotated video, or the background images themselves, and changing the background or filtering settings immediately updates the images displayed. All time-consuming operations include a progress bar indicating the approximate time to completion. Final data, including all transformations described above except for the stride calculations, are performed automatically upon export. Position data are exported in MATLAB format, and can be further analyzed using a toolbox provided in that language.

## Results

In order to examine the benefits of our tracking system, we analyzed high-speed video of tethered, walking cockroaches to extract three-dimensional patterns of leg movement simultaneously from all joints of all six legs over many strides (Fig. 6). These data were collected in “bouts” of walking, where each bout ranged from 5–8 s of continuous, forward walking at a constant speed. The maximum duration of 8 s per bout was constrained by the video recording hardware. We found that 3D patterns of joint motion are essential to understanding the patterns of control in these high-degree-of-freedom legs, especially where multiple joints can produce similar movements of the feet. Additionally, we show in detail that delimiting strides by foot anterior/posterior extreme positions (AEP/PEP, respectively) or by ground contact give different results, though both are important to understanding the control of the stepping cycle.

### Joint kinematics

**Figure 10:**
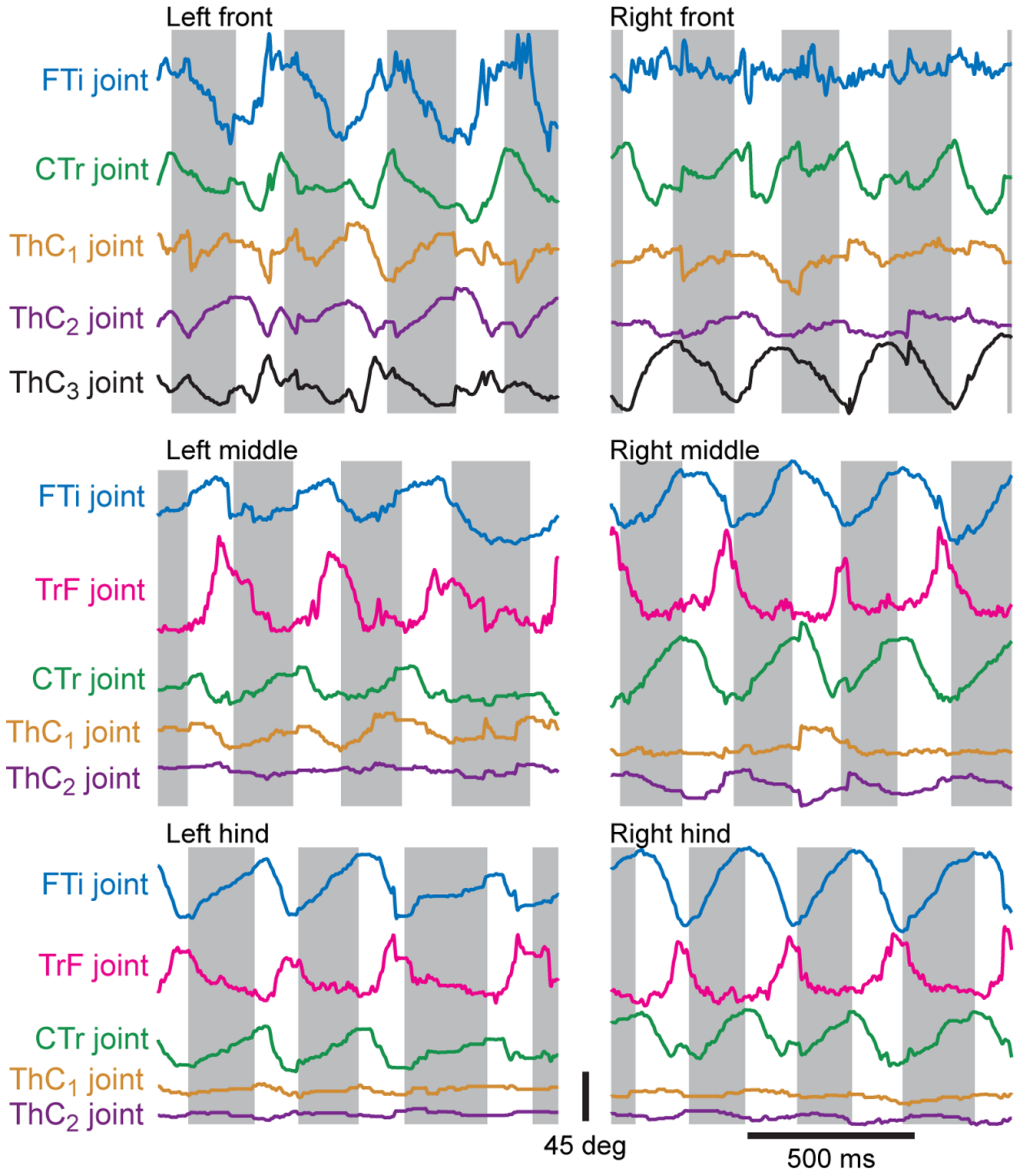
Joint angles calculated from 3D joint positions during a portion of the same walking bout shown in **Fig. 5**. As in Fig. 8, the colored points represent successive frames of tracked video. The gray boxes indicate the stance phase of each stride, defined using the *z*-position of the TiTa point. The FTi joint angle (blue) was estimated using the CTr, FTi, and TiTa points. The TrF joint angle (pink) was estimated using the CTr-FTi-TiTa plane and the coxal plane. The CTr angle (black) was estimated using the ThC, CTr, and FTi points. The angle of the coxa in the *xz*-plane centered at the ThC position was used to define the motion in the first degree of freedom (promotion/remotion) of the ThC joint (ThC_1_, green), and the declination of the coxa from the *xy*-plane determined its motion in the ThC joint's second degree of freedom (ThC_2_, orange).

It is important not to lose sight of the fact that the insect nervous system is probably not measuring or controlling the three-dimensional coordinates of the leg endpoints, but rather muscle tensions, skeletal stresses, and joint angles. A forward-kinematics computation could allow the animal to calculate 3D positions from those variables, but the 3D coordinates are not the immediately available sensory signals and are therefore probably not the controlled parameters for most behaviors. We used the leg position data to calculate three-dimensional angles for each of the leg joints other than the TiTa joint (Fig. 10). For the middle and hind legs, most of the action responsible for driving the foot through its cycle of motion comes from the femur-tibia (FTi) and coxa-trochanter (CTr) joints [18]. Running cockroaches hold their legs at an acute angle to the substrate, pointing backward along the body and nearly parallel to the abdomen. The femur and tibia are basically coplanar with the coxa, and therefore the FTi and CTr joints act to move the leg mainly forward and backward, but also lift the foot off of the ground as they bring it forward. In order to make contact with the substrate at the anterior extreme position of the leg (at the end of the swing phase), the leg must be rotated. This rotation can be done about the thorax-coxa (ThC) joint, but our data suggest that for the middle and hind legs, the CTr point (and therefore the ThC joint) move very little during forward walking (Figs. 6,10). The extreme angle of the coxa means that a small promotor/remotor movement would only move the foot slightly forward and backward (by approximately the sine of the ThC joint excursion). However, the joint between the trochanter and femur, though fused in insects with a more upright posture like stick insects and orthopterans, is actuated in cockroaches and has been shown to contribute to climbing movements [19], [20]. Fig. 10 shows that during normal walking, the TrF joints are very important in the movements of the middle and hind legs, but not so much in the front legs. The front legs have an additional degree of freedom for rotation around the ThC joint, however, and the ThC joints are in general much more active than in the other two leg pairs. Thus, the rotation of the leg is performed by the ThC joint for the front legs but by the TrF joints for the middle and hind legs. This distinction, though potentially critical for dissecting mechanisms of control in the legs, could be lost if individual legs or joints were only studied in isolation or together but in 2D, and no previous study has examined this issue.

### Stride parameters

**Figure 11:**
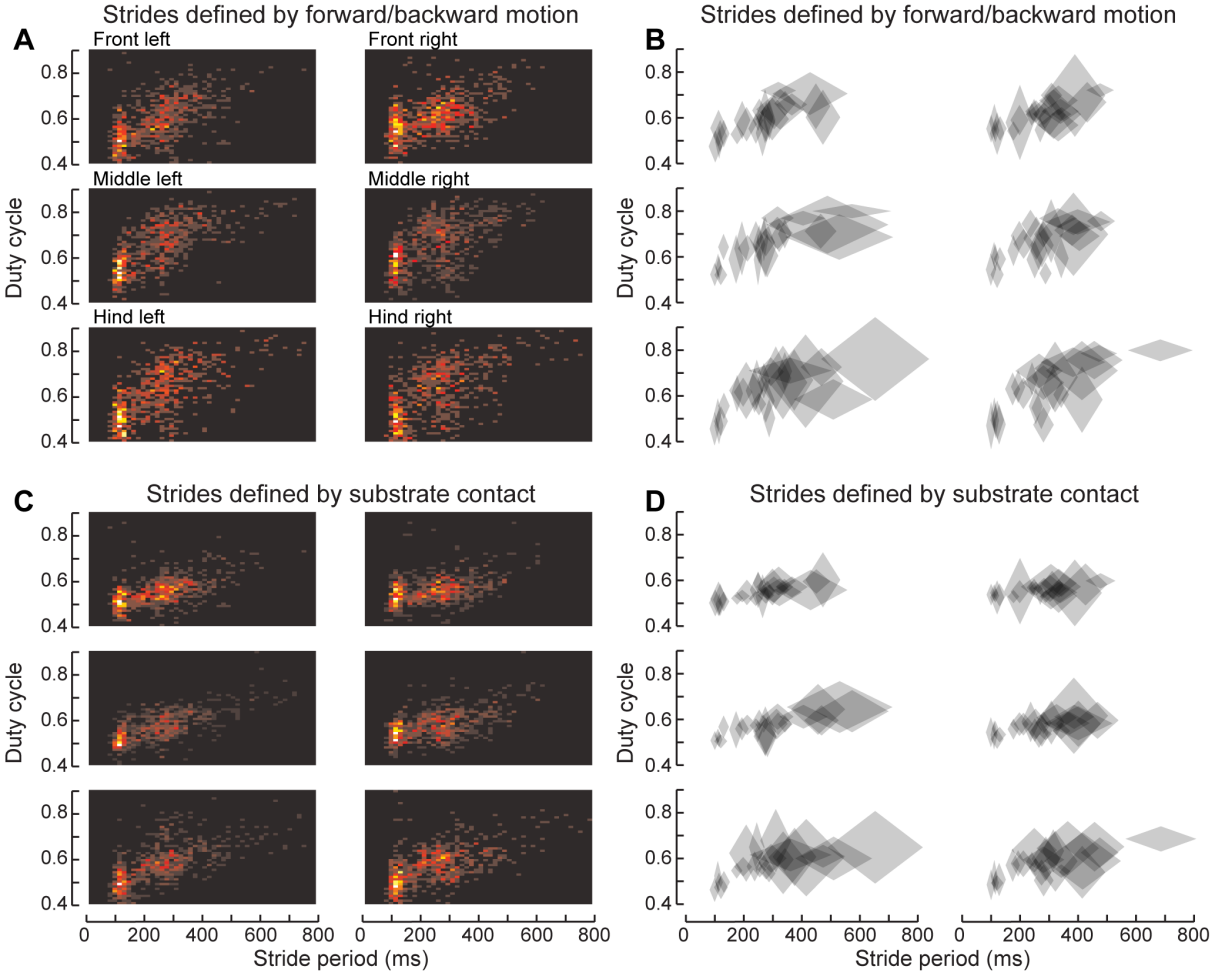
The duty cycle of the feet changes with walking speed. (A) 2D histogram of the duty cycle for each stride, plotted versus that stride's duration. Warmer colors indicate more strides in a given bin (total 763 strides from 25 bouts of walking by 8 animals). The duty cycle indicates the fraction of each stride during which the foot was on the ground, and generally decreases with increasing speed (or decreasing period). Here, the stance phase was calculated by AEP and PEP, as for the green boxes in Fig. 8. (B) Duty cycle, pooled by trial. Each diamond represents the mean and s.e.m. of both stride period and duty cycle for a single bout of walking. (C) Same data as panel A, but with stance phase determined by the TiTa points' *z*-values, as for the gray boxes in Fig. 8. (D) Same as panel B, but using *z*-values to delineate strides.

Although the three-dimensional positions we collected from multiple points on the leg gave us many additional facets of coordination to analyze, we choose here to dwell only on the transitions between swing and stance for comparison with earlier work. We used two methods to kinematically delineate periods of swing and stance, which are normally defined by whether the leg is touching the substrate. First, we approximated the transitions between swing and stance using the posterior and anterior extreme positions, respectively (PEP and AEP), of the tibia-tarsus (TiTa) joints on each leg. Second, we used the distribution of the TiTa points' *z*-values (their height above the substrate) to estimate when the leg picked up or touched down. These values yielded slightly different timing for each stride (Fig. 11), and we have plotted both because it is not clear which is more true to the cyclic interactions between muscle contractions and ground forces which physiologically define the steps. We could readily perform this comparison using our 3D data, an advantage which previous studies have lacked. Most earlier investigators measured strides using leg protraction and retraction (AEP and PEP) only, presumably because most kinematic studies have been performed in two dimensions or using rudimentary 3D analysis [1], [7], [8], [9]. Other studies using ground-reaction forces measured on a force platform or other physical measurement would probably be more comparable to our *z*-value measure of stepping [21], [22], therefore giving us the novel opportunity to bridge between the two experimental conditions.

We found that the duty cycle, the fraction of each stride spent in the stance phase, tended to decrease with increasing step frequency, though perhaps not continuously (Fig. 11). On the oiled plate, it may be possible for the legs to step at different rates if they are not synchronized centrally. However, the mean stride duration in each leg tended to be comparable within each bout of walking, except for the slowest speeds, at which the hind legs became slower and more variable in their step patterns than the other legs. The diamond symbols in Fig. 10B,D represent the mean and s.d. of both stride duration and duty cycle within each walking bout (24 total bouts from 8 animals). When the strides were dilineated by AEP and PEP, the swing phases tended to begin later and the stance phases earlier, leading to a larger duty cycle compared to strides defined by *z*-values. The differences in swing- and stance-initiation timing between each of the stride-calculation methods were exponentially distributed, with a median difference of 10–15 ms or 0.05–0.08 phase units in stance duration (data not shown). The overall differences in step period between each method followed a symmetric exponential distribution with a mean of 0. This distinction may be important for future studies on leg coordination in cockroaches, a widely studied animal for the control of locomotion.

### Speed and accuracy

The most common method of digitizing video data is for a human user to manually select the points of interest in each camera view, in each frame, with possibly some interpolation or simple 2D tracking to reduce the number of frames the user must analyze. We quantified the reliability of our tracking method by manually digitizing one 8-s movie, or 106,496 three-dimensional positions. This task required 25 man-hours of point-and-click labor, even using 2D interpolation to reduce the number of analyzed frames by 75%. By contrast, using our software package to extract these positions took around 12 hours of computer time, but only an aggregate of about 3 man-hours of labor. An infinitely fast computer would therefore reduce the total labor to 3 hours per movie, but the machine used for this study left the user with an additional ∼9 hours of nominally unused time. CPU time was on a low-end, modern desktop computer, a 64-bit Intel Core 2 Duo 4300 clocked at 1.8 GHz over a 200 MHz bus with a 2 MiB cache and 4 GiB of memory clocked at 533 MHz. Because of the parallelizable nature of our algorithm, by working forward and backward in movie time simultaneously on each of the machine's two CPU cores, our measured 12 hours of processing actually represent closer to 24 hours of CPU time.

Our software's average tracking error relative to the human “gold standard” was generally less than about 1 mm, except for the TiTa points of the front legs, which were slightly worse (Fig. 4). Some of this error could be due to the relatively low resolution of our video images (3 pixels mm^−1^). In theory, this effect could be quantified by repeatedly digitizing the same sequence by hand and calculating the variance of the points' positions across all the repetitions. A comparison of this manual-tracking precision with our software's tracking error might show that the digitization error is actually constrained by the cameras, not our tracking program. Although potentially valuable, such an analysis is unfeasible for practical reasons, given the amount of labor required to manually digitize a video.

## Discussion

Many parameters of insect walking have remained unanalyzed by previous investigators due to the difficulties of collecting the large kinematic datasets necessary to unravel the complicated mechanics of these overactuated systems. Early investigations were largely limited to describing the roles of various muscles and sensors in locomotion by relationship to the swing and stance phases of the step cycle [8], [23], [24], [25], [26], [27], [28], rather than directly to the joints. Even the large, recent expansion of knowledge regarding the interconnections between the sensory organs and the motor neurons and muscles (for review, see [29]) still lacks the critical component of joint kinematics to close the feedback loop. In other words, we are beginning to understand how sensory events affect motor neurons during walking, but we have almost no idea about the normal ways in which muscular contractions produce motion and further sensory feedback. Even the studies that have been performed were generally restricted to one leg at a time and specifically avoided the complexity of the multiplanar motions by the cockroach front legs [2], [30], [31].

Our new technique has enabled us to bring statistical power to bear by describing many consecutive strides in the same animals. Moreover, by performing this analysis in three dimensions, ideally in combination with electromyographic recordings, we can now shed light on how the muscles and joints act to control foot position and force production. Without these data, the role of the nervous system during locomotion can only be described in the abstract. Importantly, we can also compare the motions of the front, middle, and hind legs simultaneously, since an altered pattern of motion in one leg studied in isolation may or may not be evidence of a global modification. The high spatiotemporal resolution allows us to quantify subtle changes in joint motion, which will eventually be correlated with neural recordings in this preparation [30], [32], [33].

### The trochanter-femur joint

The action of the trochanter-femur (TrF) joint has been neglected in the study of insect locomotion, partially because well-studied preparations like the locust and the stick insect have a fused TrF joint, and partially because the trochanter is very small and difficult to observe. For the first time, our software has allowed a detailed analysis of its use in forward walking. In the cockroach, the TrF joint rotates along an axis parallel to the plane of the coxa, but this axis is inclined with respect to the femur by approximately 45° such that its dorsal end nearly touches the CTr joint while its ventral end is displaced laterally along the femur (see Fig. 1A). There is only one muscle activating the TrF joint, the reductor femoris [19], [34], which acts to pull the femur dorsally, toward the body. In the most common standing and walking poses, this motion acts as a supination, deflecting the tarsus ventrally toward the substrate [20]. Our data suggest that this muscle is likely to be strongly activated near the initiation of the stance phase, as the foot reaches its anterior extreme position. Presumably, the reductor femoris is therefore pre-strained as the foot touches down, and it can then act as a strut or gimbal to reduce bouncing as the body's weight shifts forward over the leg [35]. Shortly after the end of stance, the TrF joint returns to rest, presumably due to relaxation of the reductor femoris. This action will lift the tibia off the ground to commence swing. Indeed, video viewed from the side during walking shows this tibial rotation even in the absence of rotation at the coxa (Fig. 7). As noted above, the only way the tibia can be lifted without coxal rotation is through the action of the TrF joint.

These joint motions have not been quantified before during forward walking, since previous investigators lacked the resolution to discriminate TrF from ThC motion over many strides. Interestingly, it appears that the front legs of *Blaberus discoidalis* do not utilize the TrF joint. Instead, they utilize a third degree of freedom at the ThC joint, allowing the coxa to rotate around its long axis and thereby moving the tarsus through an arc in the ground plane (Fig. 10). This distinction begs further study – for example, are the muscles actuating these joints homologous, or have different muscles assumed similar functional roles? What are the implications for evolution from earlier cockroaches, such as *Periplaneta americana*, in which the front legs are shaped more like the middle and hind legs? How does local control differ in the segmental thoracic ganglia to accommodate these distinct but simultaneously occurring actions?

### Tracking software

Motion analysis using high-speed video is almost always a tedious and time-consuming task. Even worse, it is often subjective. The most commonly used method of digitizing video data is for a human user to manually select the points of interest in each frame, in each camera view. Although human digitization is slightly more accurate, the “hybrid” algorithm presented here is a compromise between objectivity and accuracy, and represents an initial step toward fully automated tracking in the future. For example, once the joint rotation axes and range of motion can be defined over a wide range of behaviors, these can be used to further constrain the 3D search for more distal leg points. Even before that, particle filtering and probabilistic assemblies such as simulated annealing could potentially be used, especially if Moore's law of processing power continues cheapen CPU cycles. Training data for more complicated models does not yet exist other than for the stick insect and the locust, but could become available for any insect through techniques such as ours.

Insect leg tracking is at best a specialized niche in the motion capture market, and commercial technologies will be slow to become applicable. For example, human pose estimation is extremely well studied [36], [37], but only transfers partially to insects because of the tight working space, leading to frequent limb occlusions, and because of the many redundant degrees of freedom in the insect leg. Tracking a cockroach's leg is roughly equivalent to tracking a human's arm joints through the fingertips, if the human had six arms and no legs. Scaling becomes an issue, too, as the physical size of the insect leg constrains the types and relative sizes of markers that can be used.

At the moment, the error rate represented here seems a reasonable price to pay for the extraction of 212,992 individual two-dimensional points to be fully accomplished in hours instead of days. This program, and its graphical user interface, was written using open-source tools in the language Python and is freely available upon request. We encourage collaborative enhancements which will be of great benefit to animal behavior research and related fields in the years to come.
